# From Traditional Breeding to Genome Editing for Boosting Productivity of the Ancient Grain Tef [*Eragrostis tef* (Zucc.) Trotter]

**DOI:** 10.3390/plants10040628

**Published:** 2021-03-25

**Authors:** Muhammad Numan, Abdul Latif Khan, Sajjad Asaf, Mohammad Salehin, Getu Beyene, Zerihun Tadele, Ayalew Ligaba-Osena

**Affiliations:** 1 Laboratory of Molecular Biology and Biotechnology, Department of Biology, University of North Carolina at Greensboro, Greensboro, NC 27412, USA; m_numan@uncg.edu (M.N.); m_salehin@uncg.edu (M.S.); 2 Natural and Medical Sciences Research Center, Biotechnology and OMICs Laboratory, University of Nizwa, Nizwa 616, Oman; abdullatif@unizwa.edu.om (A.L.K.); sajjadasaf@unizwa.edu.om (S.A.); 3 Donald Danforth Plant Science Center, St. Louis, MO 63132, USA; GDuguma@danforthcenter.org; 4 Institute of Plant Sciences, University of Bern, Altenbergrain 21, CH-3013 Bern, Switzerland; zerihun.tadele@ips.unibe.ch

**Keywords:** CRSIPR-Cas, drought tolerance, *Eragrostis tef*, genome editing, stress resilience

## Abstract

Tef (*Eragrostis tef* (Zucc.) Trotter) is a staple food crop for 70% of the Ethiopian population and is currently cultivated in several countries for grain and forage production. It is one of the most nutritious grains, and is also more resilient to marginal soil and climate conditions than major cereals such as maize, wheat and rice. However, tef is an extremely low-yielding crop, mainly due to lodging, which is when stalks fall on the ground irreversibly, and prolonged drought during the growing season. Climate change is triggering several biotic and abiotic stresses which are expected to cause severe food shortages in the foreseeable future. This has necessitated an alternative and robust approach in order to improve resilience to diverse types of stresses and increase crop yields. Traditional breeding has been extensively implemented to develop crop varieties with traits of interest, although the technique has several limitations. Currently, genome editing technologies are receiving increased interest among plant biologists as a means of improving key agronomic traits. In this review, the potential application of clustered regularly interspaced short palindromic repeats (CRISPR) and CRISPR-associated proteins (CRISPR-Cas) technology in improving stress resilience in tef is discussed. Several putative abiotic stress-resilient genes of the related monocot plant species have been discussed and proposed as target genes for editing in tef through the CRISPR-Cas system. This is expected to improve stress resilience and boost productivity, thereby ensuring food and nutrition security in the region where it is needed the most.

## 1. Introduction

The world population is increasing at an alarming rate, demanding an increase in food production. The Green Revolution of the 1960s has led to a substantial increase in major cereal production, but that is unlikely to meet the urgent demand for higher food production [1] under the current climate scenario. To meet world food demands, the production of major crops alone is insufficient, as they are less suited to extreme climate and low-input conditions [2]. There is an increasing interest in underutilized crops such as tef (*Eragrostis tef* (Zucc.) Trotter); millets, including proso millet (*Panicum miliaceum* Mill.) and finger millet (*Eleusine coracana* Gaertn.); and quinoa (*Chenopodium quinoa* Willd.), which are more versatile due to their resilience to marginal growing conditions, and outstanding nutritional values. Despite its valuable traits, the grain yield of tef is very low. In 2018, the average yield of tef in Ethiopia was only 1.7 ton ha^−1^ as compared to maize (4 ton ha^−1^) and wheat (2.7 ton ha^−1^) [3]. Tef is a cereal crop originating in the Horn of Africa, which is widely cultivated in Ethiopia and Eritrea. In Ethiopia, tef is a staple food for about 70% of the population. The crop is annually cultivated on 2.9 million hectares of land, producing about 4.5 million tons of grain [4]. Tef is tolerant to marginal soil and unfavorable climate conditions, which makes it a potential crop for arid and semiarid areas as well as poorly drained soils [5]. Tef is also one of the most nutrient-dense crops, containing high amounts of macro- and micro-nutrients (primarily calcium and iron), amino acids and vitamins [6]. Tef cultivation in Ethiopia and around the globe has increased in recent years due to its many health-related benefits. Since the absence of gluten epitopes has been confirmed in tef by antibody assays [7], it has been recommended as an alternative diet for people suffering from celiac disease, the immune reaction to consuming gluten containing foods such as wheat (*Triticum aestivum* L.), barley (*Hordeum vulgare* L.) and rye (*Secale cereal* L.), which affects 0.6–1.0 percent of the population globally [8,9]. In addition to the extensive use of tef grain for human consumption, the straw of tef is more nutritious and palatable as a livestock feed compared to the straw from cereals such as barley and wheat [10]. Moreover, tef straw is used as construction material because it serves as an organic binder for mud used for plastering walls for local houses [11]. Various agronomic traits, such as panicle architecture, tilling, grain size and plant height, have been targets for the improvement of tef yield. Grain yield is a highly complex trait which has several components, including seed weight, form and size of panicles, florets per panicle and number of fertile tillers [12,13]. Other important traits that determine grain yield include shoot biomass, panicle weight and the number of tillers in a plant [14]. Furthermore, certain agronomic traits such as shattering proneness, lodging tolerance, dry matter yield, leaf area, and plant height directly or indirectly influence grain yield in crops [15,16].

The main factors causing yield loss in tef include susceptibility to lodging, weed competition, drought, small grain size and soil acidity [5]. Although tef shows several agronomic and nutritionally desirable traits, it is under tremendous pressure due to harsh environmental stress conditions [5]. The crop is relatively resistant to diseases and insect pests as compared to other cereal crops. Among abiotic stressors, tef yield is significantly reduced by drought and soil acidity. Weed competition has broad about a range of effects on the yield of tef in Ethiopia [17]. Many direct and indirect strategies of weed control are employed by farmers [18]. Hand weeding and frequent tillage are the two commonly used methods of weed control in tef production. Furthermore, weeds can be controlled by herbicide application with proper management of spray times and frequency. However, the herbicides must be specific to broad-leaved weeds to avoid damaging tef plants. Taken together, hand weeding, the use of herbicides and resistant tef varieties are viable alternatives in order to overcome yield loss due to weeds. With proper weed control methods, improved tef varieties such as *Kora* and *Quncho* have been shown to produce higher yields [19].

Drought is a major abiotic stress which has significant effects on crop yield in most African countries. Water scarcity has resulted in a fragile ecosystem in Africa’s arid and semiarid regions. In sub-Saharan Africa, about 1.1 billion people live in drier environments; however, this number is expected to double by 2050, and is expected to reach 2.5 billion people [20]. Drought stress after planting [21] and during the flowering and grain filling stage has serious effects on crop yields, and up to 60% of yield loss has been reported in pearl millet at these stages [21,22]. In tef, drought has been reported to cause about 40% yield loss [23].

The other major cause of low productivity in tef is lodging, which is the displacement of the stalks from the vertical position due to wind and rain [24]. Lodging occurs frequently before grain maturity, significantly reducing the grain yield [25]. Tef is primarily susceptible to stem lodging [26,27]. Panicle length is also associated with lodging tolerance [25]. Semi-dwarf varieties of tef are lodging-tolerant and produce higher yields than tall varieties [28]. Lodging limits the use of inputs such as N-fertilizers, exacerbating the susceptibility of the plant to lodging [29].

To overcome the effects of the constrains mentioned above and to improve the tef productivity, it is important to develop resistant and high-yield verities. There are several approaches to increasing crop productivity as well as stress tolerance in crops. Among these strategies, genome editing techniques have recently received increased attention. Previous studies have suggested that the productivity of many cereal crops such as maize [30,31], rice [32,33,34,35], wheat [30,36] and other monocots [37,38] have been improved using the clustered regularly interspaced short palindromic repeats (CRISPR) system. In rice (*Oryza sativa* L.), CRISPR-associated proteins (CRISPR-Cas) systems have been used to improve tolerance to drought [39], cold [40] and salt stress [41,42], ultimately boosting productivity [39]. In wheat, two efficient and simple CRISPR-Cas methods have been developed to improve productivity and stress resilience [43,44,45]. The CRISPR-Cas technology used in these monocots is expected to be transferred to tef. Therefore, the aim of this review is to highlight the potential of CRISPR-Cas-mediated gene-editing in trait improvement in tef.

## 2. Mechanisms of Tolerance to Lodging and Environmental Constraints in Tef

### 2.1. Lodging Tolerance 

Lodging is the process by which cereal shoots are displaced from an upright position to a horizontal position [46]. Lodging is considered a complex phenomenon, influenced by several factors, such as diseases, agronomic practice, crop history, soil type, landscape, geography, rain and wind [47]. Stem lodging is the bending or breaking of stem internodes (lower culm internodes), whereas root lodging is the failure of the root to maintain its integrity in the soil [48]. The application of fertilizers aggravates lodging, and hence the yield potential of tef. Lodging stress can be reduced by controlling/decreasing plant height. However, reducing plant height by inhibiting plant growth regulators or introducing dwarfing genes could lead to crop yield reductions [47]; hence, researchers have suggested targeting traits other than plant height to reduce yield loss due to lodging. A recent study by Merchuk-Ovnat, et al. [49] suggested that early lodging is likely caused by a rapid increase in inflorescence weight [49]. This group also observed variations among the tested tef population in terms of lodging time and strength, with some populations possessing the strength to hold the inflorescence in the grain filling season up to a certain point before they were bent to the ground. Due to its weak stem, tef has high chance of succumbing to lodging due to rain or wind [50]. Modification of the stem’s chemical composition, such as its cellulose, lignin, structural carbohydrate and silica composition, is expected to increase lodging-, disease-, and pest-resistance [51]. Silicon (Si) is a beneficial plant nutrient that has been shown to increase tolerance to lodging, diseases and pests, as well as to abiotic stresses such as drought, salinity, heavy metal stresses, and extreme temperature in various crops, ultimately leading to increased grain yield [52,53,54,55,56]. We recently performed greenhouse experiments to study whether tef benefits from Si application. Our findings revealed that Si improves grain and biomass yield, stress resilience, and regulates the expression of Si-transporter genes in tef [57]. However, conclusive evidence showing the mechanism of silicon-induced stress resilience is lacking [58].

Although lodging is the main cause of low yield in tef [59], both physiological and molecular aspects are understudied, and biotechnological, molecular and breeding techniques [47] are not well developed to prevent lodging. A partnership formed by the ‘Tef Improvement Project’ has recently developed semi-dwarf and lodging-tolerant tef varieties, which are currently being disseminated in farmer’s fields in Ethiopia [60].

Lodging tolerance has been shown to be improved by modulating the biosynthesis of plant growth regulators (PGRs). For example, the inhibition of gibberellic acid (GA) has been shown to reduce plant height [46,61] and decrease lodging susceptibility. Shorter internodes are associated with reduced plant height [62]. During the Green Revolution of the 1960s and 1970s, inhibition or alteration of GA in rice and wheat was mainly targeted for developing semi-dwarf varieties, which ultimately boosted the yield of these crops [63]. In tef, mutation in the *α-Tubulin* gene is associated with agronomically important traits such as semi-dwarfism and lodging tolerance [59]. Blösch, et al. [25] have reported that panicle angle contributes to lodging tolerance in tef. Jifar, et al. [28] also identified some lodging tolerance genotypes (*RIL-91*, *RIL-244* and *RIL-11*).

Genes associated with dwarfism in plants have been widely studied [64,65,66,67,68,69]. The two prominent genes of the 1960s Green Revolution were the semi-dwarf (*SD1*) gene in rice [66,70,71] and reduced height-1 (*RHT-B1b* and *RHT-D1b*) in wheat [72]. SD1 belongs to the gibberellin biosynthetic pathway, whereas *RHT* is a GA response regulator and is a *DELLA* protein family gene. *DELLA* proteins are important components of the signal transduction pathway of GA, encoded by the wild-type allele of *RHT-B1b* and *RHT-D1b* [73]. In the Green Revolution wheat varieties, introduction of a stop codon in the N-terminus of the two reduced height-1 (*RHT-B1* and *RHT-D1*) loci was responsible for the semi-dwarf and lodging tolerance traits [72]. In rice, the enzyme gibberellin 20-oxidase (*GA20*) encoded by the *SD1* gene is responsible for the biosynthesis of GA [65,74]. A frame shift mutation due to a 383-bp deletion in the *sd1* allele has been shown to greatly reduce the level of *GA20* oxidase [66]. Mutation of the *sd1* and *RHT* homologs in tef could potentially lead to lodging tolerance and significantly improve grain yield. Similarly, genetic loci (*DW1*, *DW2*, *DW3* and *DW4*) that control plant height across several environmental conditions have been identified in sorghum. Recently, scientists have transferred these mutations into a single sorghum line and managed to release a semi-dwarf commercial variety that contains mutations in three loci (*DW1*, *DW2* and *DW4*) [75,76]. This suggests that these mutations could also be introduced into tef to develop semi-dwarf varieties with improved stress tolerance and enhanced grain yield.

### 2.2. Drought Tolerance

Understanding the degree of stress tolerance in crop plants is important in devising alternative strategies for improving yield and quality. Drought is one of the most important abiotic stresses affecting plant growth and development. Plants have developed various mechanisms of drought tolerance [77,78]. The mechanisms that have been reported in tef include modifications of stomatal conductance, osmotic adjustment, development of a deep rooting system and maintenance of cell membrane stability [79,80]. Development of a deep root system and osmotic adjustment are major drought stress tolerance mechanisms in many crops, including tef [79]. The association of plant height, root depth and thickness to drought stress tolerance was previously reported in tef [79]. Recently, crosstalk between plant height and drought tolerance was reported from a study on tef and other small cereals where semi-dwarf plants were found to be drought-tolerant [81]. Osmotic adjustment is also known to enable tef leaves to maintain leaf turgor pressure (LTP) [79,82] under extreme drought conditions by retrieving and absorbing water even from dry soils. Modification of root growth parameters in response to water scarcity is another strategy used to mitigate drought stress [83,84]. For example, the increase in root length of cowpea, peanut and soybean plants when exposed to drought enabled them to absorb deep soil water [84]. Similarly, developing deep-rooted tef plants with an extensive and broad root system is a desirable trait to withstand drought stress [79].

### 2.3. Weed Competition and Herbicide Tolerance

Weed competition is another important plant trait in areas of low-input integrated weed management systems [85]. The competitive ability of crops has been divided into two broad categories; the first category is the crop’s ability to reduce competitor fitness, whereas the second is the crop’s ability to resist yield losses and withstand its neighbor’s competitive impact [86]. Different terms have been used for these aspects in the literature, such as “tolerance ability” and “suppressive ability” [87,88].

In Ethiopia, smallholder farmers have adopted some cultural methods to mitigate the impact of weed competition. Hand weeding and frequent tillage are common practices used to control weeds in tef production [17]. Herbicides are not widely used, mainly due to economic reasons and shortages of supplies. An alternative strategy in weed management is the use of cultivars with competitive ability due to their sustainability [88,89]. However, information on tef varieties with high weed competitive ability is limited as compared to other cereals such as oats (*Avena sativa* L.), barley (*Hordeum vulgare* L.) and wheat (*Triticum aestivum* L.) [86]. Tef varieties can be improved using genetic modification tools such as the CRISPR system to improve weed tolerance and enhance productivity. Potential genes for weed resistance and yield improvement can be overexpressed in tef or engineered through the CRISPR-Cas system to minimize the impact of weed competition.

Herbicide-resistant varieties have been developed in crops such as soybean by targeting key genes in amino acid synthesis or other functions. Among these genes, acetolactate synthase (ALS) is involved in the synthesis of branched-chain amino acids such as isoleucine, leucine and valine [90]. ALS is the target site for five non-competitive inhibitor families—sulfonylaminocarbonyltriazolinones, pyrimidinylthiobenzoates, triazolopyrimidines, imidazolinones and sulfonylureas [91]. Plants engineered in the *ALS* gene are resistant to non-selective herbicides, whereas all non-engineered plants, including weeds, are sensitive to the non-selective herbicides. A similar principle was implemented to develop glyphosate-resistant plants in which the *EPSPS* (*5-enolpyruvylshikimate-3-phosphate synthase*) gene was targeted. The *EPSPS* gene is involved in the shikimate cycle [92]. Overexpression or knockout of the above-mentioned genes might contribute towards developing tef plants with resistance to non-selective herbicides.

### 2.4. Panicle Architecture

Panicle architecture and grain size are important yield traits in cereal crops such as rice, wheat and barley [93,94,95]. There is a direct relationship between agronomic traits such as panicle number, number of spikelets in panicle, spikelet filling percentage, grain size and number and crop yield [96]. For example, in rice, higher grain yield in a hybrid variety is associated with the number of spikelets in a panicle [96,97]. In some crops, genes that control panicle number and grain size have been identified and modified to increase yield [98,99,100]. For example, *OsSPL14* (squamosa promoter binding protein-like 14) gene and microRNA “*OsmiR397*” promoted panicle branching and increased grain size in rice, which ultimately lead to high grain yield [99,101]. In tef, homologs of these genes remain to be identified and characterized to determine their role in increasing grain size and to improve yield.

## 3. Status of Tef Improvement

### 3.1. Traditional Breeding: Past and Current Status of Tef Improvement

**Figure 1:**
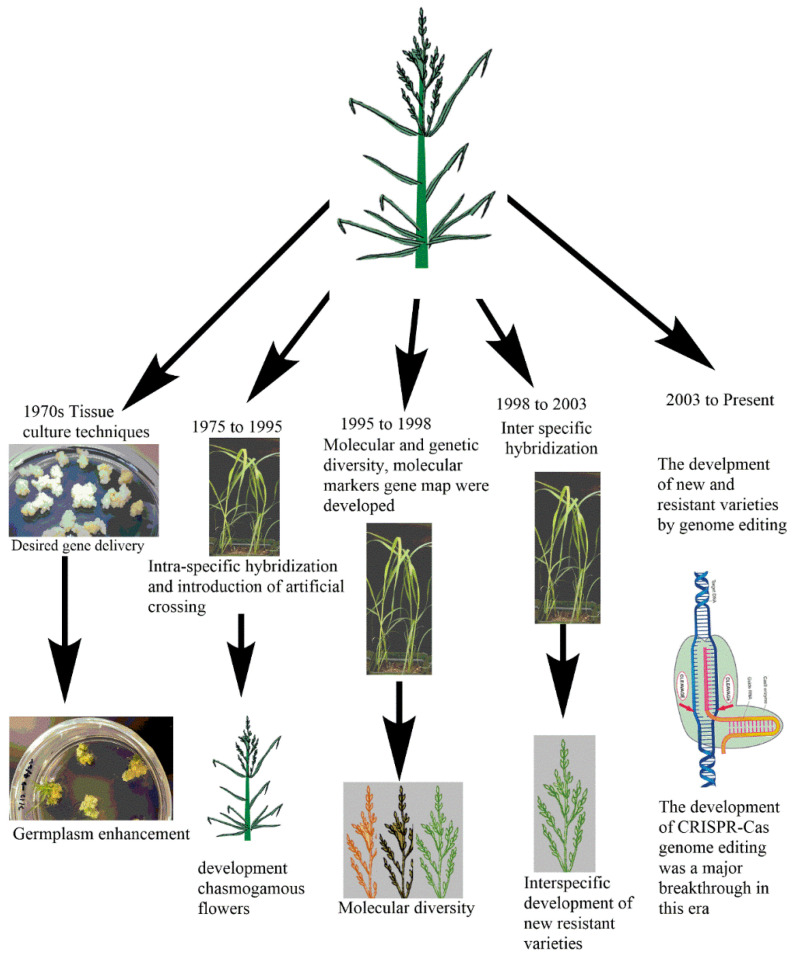
Improvement of tef varieties over the last 50 years. The improvement of tef started back in 1970s with tissue culture techniques, followed by hybridization, the study of molecular diversity, molecular marker analysis, the development of resistant varieties by interspecific hybridization and mutation and the recently emerged clustered regularly interspaced short palindromic repeats (CRISPR)-associated proteins (CRISPR-Cas) genome editing technique. Note: (The pictures used in this figure were either taken in the author’s labs or drawn using ChemBioDraw software).

Scientific research on tef started in Ethiopia in 1950s [102]. Early breeding work focused on germplasm enhancement through collection, characterization, evaluation and conservation, as well as genetic improvement in which pure lines were selected from already existing germplasm [11,103] (Figure 1). Since flower opening characteristics were revealed in tef in 1974, [104], hybridization was used as a means of tef improvement. Molecular approaches in tef including marker development, genetic linkage maps, genetic and molecular diversity analysis were initiated during 1995–1998 [11]. Further progress was made during 1998–2003, including the initiation of interspecific hybridization, in vitro culture and mutagenesis in order to improve disease and lodging resistance. Over the last two decades, there has been progress in the area of tef genetic architecture and genomics research [105,106] (Figure 1). From a total of 42 improved tef varieties released by the National Research Program in Ethiopia, 18 were developed using the hybridization technique [107].

### 3.2. Molecular Marker Development 

The application of molecular markers in tef improvement was initiated during 1995–1998 [11]. Molecular markers near target genes are utilized for marker-assisted selection (MAS) or marker-assisted breeding (MAB) [108]. They enable the effective use of alleles during the selection of phenotypes. The most commonly used markers are microsatellites (simple sequence repeats; SSRs), amplified fragment length polymorphism (AFLPs) and single nucleotide polymorphisms (SNPs) [108]. During the selection of molecular markers, some important factors are considered, such as the quality and quantity of required DNA, procedures for marker assays, the level of polymorphism and the cost of the marker [109]. In tef, the SSRs and expressed sequence tag (EST), restriction fragment length polymorphisms (RFLPs) and random amplified polymorphic DNA (RAPD) have been developed [110,111]. Through SSR analysis, Abraha, et al. [112] identified and improved some important traits in tef, including grain yield, days to maturity, panicle length and plant height. Similarly, variability in tef accessions was identified using AFLP markers, which can be used in seed multiplication and breeding programs [113]. Application of these markers could play a great role in environmental stress tolerance in tef for improved productivity. Targeting induced local lesions in genomes (TILLING) is another genetic method used to identify small deletions or single base pair changes (mutation detection) in specific target genes [114]. In tef, targeting induced local lesions in genomes (TILLING) was used for targeting and improving valuable agronomic traits such as drought tolerance, seed size and dwarfism [115].

## 4. Potential of Genome Editing Technologies for Tef Improvement

**Figure 2:**
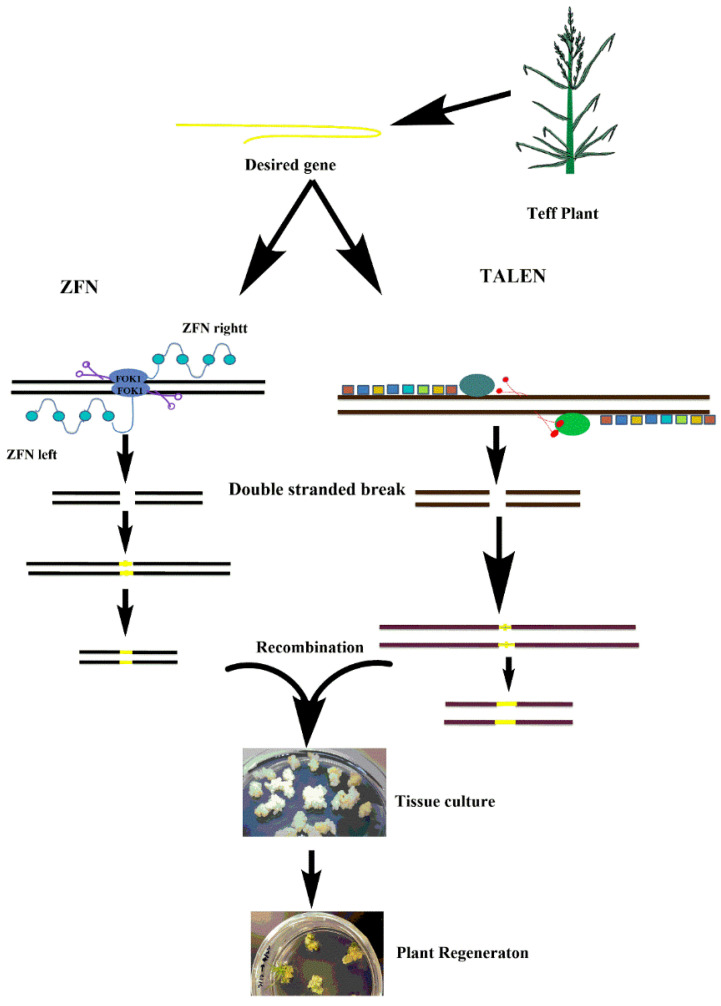
A schematic view of genome editing by zinc finger nuclease (ZFN) and transcriptional activator-like effector nuclease (TALEN) in tef. A desired gene is selected from tef and integrated with ZFN and TALEN and then transferred to a cell through a vector, which will then introduce a break into the double-stranded DNA and integrate the gene of interest into the host genome. Transformed cells are used to regenerate to whole plants. (Note: the pictures used in this figure were either taken in the author’s labs or drawn using ChemBioDraw software).

Genome editing is one of the most recently developed technologies that has great potential to improve abiotic stress tolerance and boost productivity in tef. In a given genome, DNA can be replaced, inserted or deleted at an endogenous loci through a robust genetic engineering technique using sequence-specific nucleases (SSNs) [116]. SSNs such as CRISPR and CRISPR-associated protein 9 (CRISPR-Cas9) [117,118,119,120], transcriptional activator-like effector nuclease (TALEN) [121,122,123] and zinc finger nuclease (ZFN) [124,125] have been implicated in rapid genome editing in recent years. In addition to these, plant scientists use other techniques such as base editing, prime editing [126] and CRSIPR-Cpf1 [127]. Recently, CRISPR-Cpf1 has successfully used the prime genome editing in wheat Lin, et al. [128] and rice Lin, et al. [128], Li, et al. [129] genomes. These genome editing tools have been used in model plants, but with advances in genome editing, these procedures are now customized for wide variety of plant species and are usually specific to genotype [130] (Figure 2). However, to adopt advanced genetic engineering technologies in tef, there must be a well-established transformation and regeneration system, which is currently underdeveloped or non-existent for underutilized crops including tef. Recent advances in transgenic technologies have revealed promising tools for enhancing transformation and regeneration of transgenic lines. For example, overexpression of the maize embryogenic regulator genes baby boom (*Bbm*) and Wuschel2 (*Wus2*) has been shown to produce high transformation frequencies in numerous previously non-transformable monocot species, including maize inbred lines, sorghum (*Sorghum bicolor* (L.) Moench), sugarcane (*Saccharum officinarum* L.) and indica rice (*Oryza sativa* ssp. *indica*) [131]. More recently, Debernardi et al. [132] reported that expression of a fusion protein combining wheat growth-regulating factor 4 (GRF4) and its cofactor GRF-interacting factor 1 (GIF1) has been shown to substantially increase the efficiency and speed of regeneration in wheat, triticale and rice and increase the number of transformable wheat genotypes. These approaches have great potential for genetic improvement of tef and other recalcitrant economically important crops.

Since its first application as a plant genome editing technique [120,133,134], CRISPR-Cas has been widely applied in crop improvement programs [135,136]. Major crops that have benefited from the CRISPR-Cas technique include rice [32,33,34,35], maize [30,31], wheat [30,36] and other monocots [38]. In rice (*Oryza sativa*), the CRISPR-Cas system has been used to enhance drought [39], cold [40] and salt [41,42] tolerance, and to boost productivity [39]. Recently, in wheat, which is one of the plant species that is considered recalcitrant to genetic transformation via the *Agrobacterium* method, two efficient and simple CRISPR-Cas methods were developed [43,44,45]. Taken together, CRISPR-Cas technology has been widely implemented in both monocots and dicots, and has great potential to be implemented in tef improvement so that the performance of the crop against diverse environmental stresses will be enhanced, with the ultimate goal of boosting productivity.

### Candidate Tef Genes for CRISPR-Cas Technology

**Figure 3:**
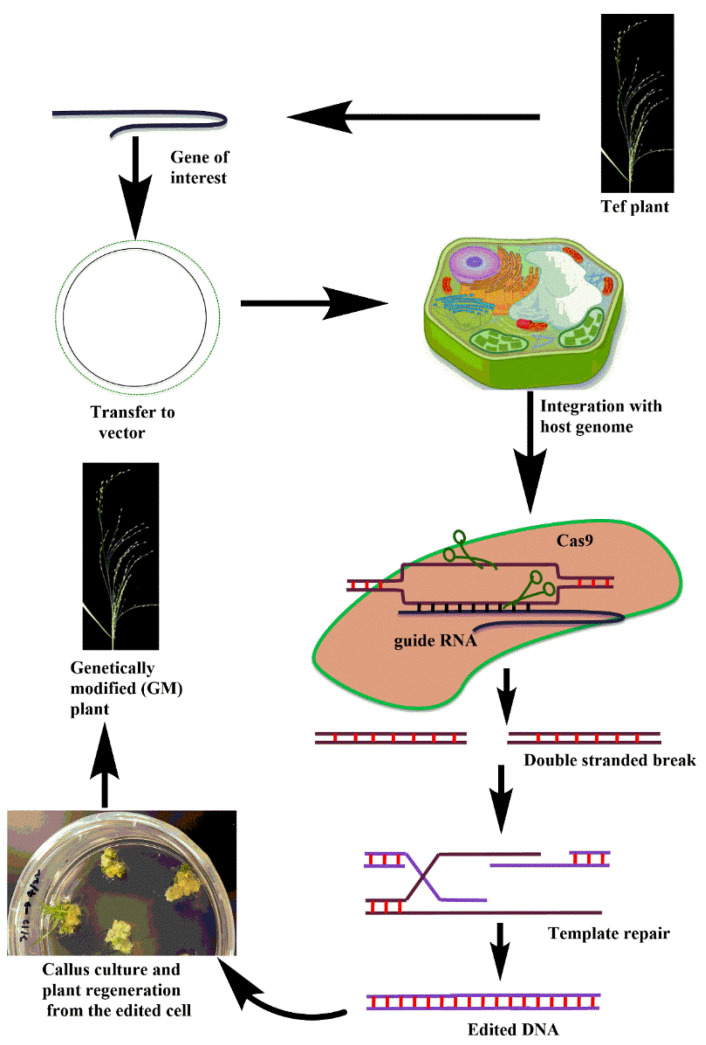
Illustration of the CRISPR-Cas system for tef genome editing. The gene of interest is transferred into a binary vector, which will be transferred into the target tissue (e.g., embryogenic calli) via *Agrobacterium* transformation, where the CRISPR-Cas protein machinery binds and breaks the double-stranded DNA of the gene of interest. CRISPR-edited lines will be regenerated from rthe callus. (Note: the pictures used in this figure were either taken in the author’s labs or drawn using ChemBioDraw software).

The CRISPR-Cas system has proven efficient because it uses a single guide RNA through pairing of DNA targeting [137,138]. Targeting of DNA is essential for genome editing across all organisms [139]. In order to edit any plant gene using the CRISPR-Cas system, it is not necessary to integrate into the genome. For example, a guide RNA and Cas can be expressed transiently in the protoplast to edit a plant genome, and the protoplast can be regenerated into whole plant. Cas is a class II CRISPR system which is used in various organisms as a gene editing tool [138,140]. The basic mechanism involved in CRISPR-Cas editing is transformation to cells, followed by its integration with the host genome, and expression, where it cuts the specific locus of interest on the chromosome. The genome cleavage requires the Cas system, together with a single guided RNA (sgRNA): fusion of trans-activating (tracr RNA) and CRISPR RNAs (crRNA), followed by the recognition of the desired DNA sequence and protospacer-adjacent motifs (PAMs) (Figure 3) [138].

**Figure 4:**
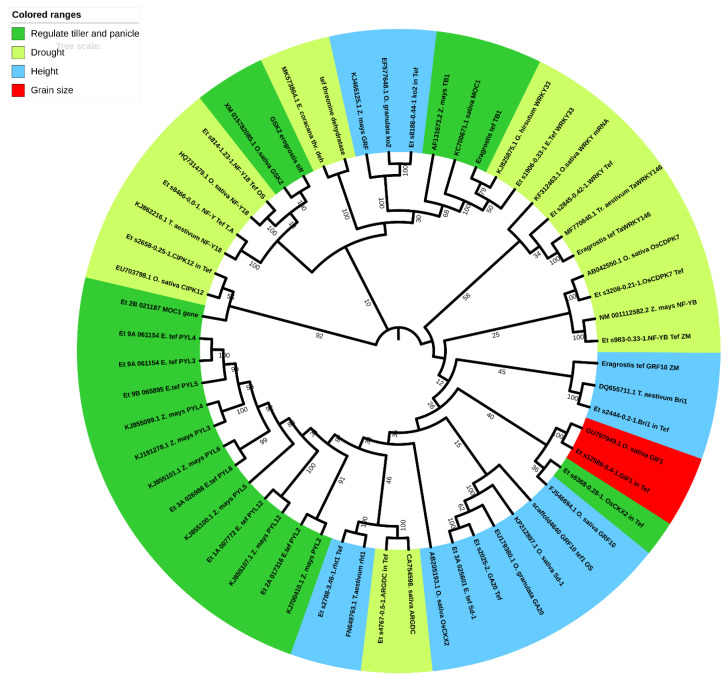
Phylogenetic tree of stress-resistant genes in tef and related monocots. The tree was constructed by using specific gene sequences downloaded from NCBI and Ensembl Plants. Bootstrap values (1000 pseudoreplicates) are shown on the nodes of the branches.

To utilize CRISPR-Cas technology in tef improvement, identification of target genes that regulate agronomically important traits is crucial. In this review, we explored the draft genome sequence of tef [141] to identify genes that are possible targets for improved yield and abiotic stress tolerance. We reviewed the literature for genes which are negative regulators of abiotic stress tolerance, and those that regulate plant height and yield attributes in monocots, including rice, maize, wheat and finger millet, which is closely related to tef. We then searched for homologs in tef (Table 1) from the Ensembl plant database using *CoGeBlast*-comparative genomics databases [142]. The tef homologs were aligned with those in other monocots using the *Mega X clustlaw* alignment tool [143,144]. After alignment, a phylogenetic tree was constructed using the *Mega X maximum likelihood* tool [144] (Figure 4). It can be observed from Figure 4 that the tef homologs showed maximum bootstrap values with those of the other monocots.

Tef is tolerant to poor soil conditions including waterlogging and drought [145]. However, tef yield is reduced by lodging, terminal drought and diseases. Therefore, tef is expected to benefit from CRISPR-Cas genome editing technology. The draft genome sequence of tef has been released [141]. Two complete homologous chromosomes with syntenic gene pairs have been reported in the tef genome due to its allotetraploid genome. The subgenomes are small (~300 Mb), with a low number of transposable elements (TE) and a high density of genes as compared to other polyploid grasses [141]. One of the major obstacles for the targeted breeding of tef is the presence of genes in two genomes (AA and BB: tef is allotetraploid, with 2n = 4x = 40 chromosomes). Gene redundancy poses a difficulty in mutagenesis for developing lodging-resistant and semi-dwarf varieties [146]. This obstacle can be overcome by techniques such as targeted genome engineering and marker assisted selection. In a plant genome, the majority of genes have variable expression patterns; therefore, the two sub-genomes are more likely to affect agronomic traits with different frequencies [141,147]. To utilize CRISPR-Cas technology in tef improvement, the identification of target genes that regulate agronomically important traits is crucial.

## 5. Constraints and Solutions Related to CRISPR-Cas Genome Editing 

The stable transfer of the transgene into the target site using CRISPR-Cas during the transformation process may cause the insertion of plasmid DNA or unwanted genes, which makes it a genetically modified (GM) crop. This limits the use of CRISPR-Cas system for sustainable agriculture and biotechnology because in some countries the use of GMOs is either tightly regulated or totally prohibited [168]. Although genetic segregation is the process by which the foreign DNA can be removed, this is not applicable to some clonally propagated plants. Moreover, in some countries, CRISPR-Cas products are still not acceptable because foreign DNA materials are used in the process, although these foreign materials are removed at the end [168]. In plants, DNA-free genome editing has been conducted using two approaches; these are pre-assembled ribonucleoproteins (RNPs) [169,170] and the delivery of a combination of guide RNA and mRNA-encoding Cas [43]. However, CRISPR-Cas RNA transient expression efficiency is low, suggesting a need for additional optimization. Following this approach, the addition of a protectant for stabilizing RNA could prove to be a promising strategy [171].

Another major drawback of the CRISPR-Cas system is its non-specificity. In this case, Cas cleaves DNA at non-target sites that are not complementary of single guide RNA [172]. This drawback impedes CRISPR-Cas potential applications, particularly when genome alteration needs to be precise, as in the case of gene therapy. Off-target sites may not change plant breeding as much as the chemical and physical alterations used in traditional breeding procedures, which generate many alterations in plants [173]. These off-target alterations can be removed by performing backcrossing to the original plant. However, this takes several generations of investigation, and the improvement of the process will be slow.

In plants, the specificity of the CRISPR-Cas system has been estimated by deliberate non-target investigation [174]. For RNPs, non-target alterations were hardly recognized by thorough sequencing, indicating that RNPs enhance the specificity of the editing system [172]. However, no study has been reported on Cas specificity in plants. Several impartial strategies which include Digenome-seq, high-throughput genome-wide translocation sequencing (HTGTS), genome-wide unbiased identification of double stranded breaks (DSBs) enabled by sequencing (GUIDE-seq) and breaks labeling, enrichment on streptavidin, and sequencing (BLESS) have been used to investigate non-specific changes in human cells [175,176,177,178], and these strategies need to be administered in plants to evaluate Cas specificity at the genome level. The need for improving its specificity is a major challenge for CRISPR-Cas genome editing, which requires attention. Various strategies have been established for improving specificity [179], including high-fidelity Cas variants and the Cas paired nickase strategy [180,181,182].

## 6. Conclusions

Climate change and global warming are expected to trigger major abiotic stresses, which are expected to reduce crop yields and ultimately lead to food shortages in the foreseeable future. Since agricultural crops fulfill most of the world’s food supply, it should be the topmost priority of plant biologists to take concrete measures to cope with climate change and the forecasted food shortages. Climate change and global warming are manifested by abiotic stress factors that could reduce crop productivity. The goal of this review was to provide an insight on the potential of advanced tools such as CRISPR-Cas for use by plant biologists in order to improve stress resilience, modify plant architecture and improve productivity. Application of this cutting-edge technology in underutilized/orphan crops such as tef will provide several benefits. It is expected to improve food security in the Horn of Africa, a region which is very vulnerable to the negative impact of climate change, and which has been experiencing frequent food insecurity and adding to the global refugee crisis. It will also enhance the acceptance of tef as a healthy and nutritious grain, which will play a role in reducing micronutrient deficiency.

## Figures and Tables

**Table 1:** Summary of genes involved in key agronomic traits of selected crops. Homologs of these genes in tef were downloaded from the genomic database to identify potential candidate genes for CRISPR-Cas-mediated gene editing in tef.

Gene	Plant Name	Accession Number	Reference
**Plant Height**			
KO_2_	*Oryza sativa Japonica*	AY660664	[148]
GA regulatory factor-like (GRF) mRNA	*Zea mays*	KJ466125	[149]
growth-regulating factor 10 (GRF10)	*Oryza sativa Indica*	FJ546694	[150]
GA20-oxidase (GA20ox2)	*Oryza granulata*	EU179380	[151]
BRI1	*Triticum aestivum*	DQ655711	[152]
Sd-1 (used in green revl)	*Oryza sativa*	KP212897.1	[70]
RHT1	*Triticum aestivum*	FN649763	[153]
**Number of Tillers and Panicle Branches**			
OsCKX2	*Oryza sativa*	AB205193.1	[154]
teosinte branched1 (tb1)	*switchgrass*	AF131673.2	[155]
GSK2	*Oryza sativa*	XM_015782085	[156]
PYL2	*Oryza sativa*	KJ700410.1	[157]
PYL3,	*Oryza sativa*	KJ191278.1	
PYL4,	*Oryza sativa*	KJ855099.1	
PYL5,	*Oryza sativa*	KJ855100.1	
PYL6	*Oryza sativa*	KJ855101.1	
PYL12	*Oryza sativa*	KJ855107.1	
monoculm1 MOC1	*Oryza sativa Japonica*	KC700671.1	[158]
**Grain Size**			
G1F1A	*Oryza sativa*	GU797949	[159]
**Drought Tolerance**			
GhWRKY33	*Gossypium hirsutum*	KJ825875.1	[160]
WRKY mRNA	*Triticum aestivum*	KT865879	[161]
threonine dehydratase mRNA	*Eleusine coracana*	MK573864	[162]
OsCDPK7	*Oryza sativa Japonica*	AB042550	[163]
TaWRKY146	*Triticum aestivum*	MF770640.1	[164]
NF-Y18	*Oryza sativa Japonica*	HQ731479	[165]
Arginine decarboxylase (ADC)	*Oryza sativa Japonica*	CA754598.1	[166]
CIPK12	*Oryza sativa Japonica*	EU703798	[166]
NF-YB	*Zea mays*	NM_001112582	[167]

## Data Availability

Not applicable.
